# Efficiencies in Physical Talent Identification Among Australian Adolescents: A Retrospective, Cross-Sectional Observational Study

**DOI:** 10.3390/jfmk11020160

**Published:** 2026-04-20

**Authors:** Patrick W. R. Norton, Stephen J. Norton, Kevin I. Norton

**Affiliations:** 1 School of Allied Health Science and Practice, Alliance for Research in Exercise, Nutrition and Activity, Adelaide University, Adelaide, SA 5005, Australia; pat.norton@adelaide.edu.au; 2 Independent Researcher, Adelaide, SA 5005, Australia; stephen@sportmatch.com.au; 3 School of Health Sciences, Faculty of Medicine and Health, University of New South Wales, Sydney, NSW 2052, Australia

**Keywords:** physical performance, sports development, talent search, adolescent athletes, anthropometry

## Abstract

**Background**: Talent identification (TID) programmes aim to detect adolescents with high physical potential, yet the efficiency of finding high-performance talent across different testing environments in an Australian context is unknown. The current study aim was to calculate the likelihood of participants scoring at or above the 90th percentile in anthropometric or physical performance measures across different testing settings. **Methods**: We analysed retrospective, cross-sectional physical and performance data from 10,134 Australian adolescents aged 12–17 years (4427 girls; 5707 boys) tested in either schools (2992; 3500), advertised come-and-try TID “Select” sessions (1235; 1622), or community-based amateur sports clubs (200; 585). Standardised measures used across all settings included height, body mass, and five physical performance tests of strength, speed, agility, leg power and aerobic fitness. We used a threshold of “higher physical performance” or “physical talent” as an age- and sex-specific ≥90th percentile ranking in any of the performance tests when compared against our international normative database. Anthropometry measures were also compared using the same approach across settings. **Results**: Chi-square tests showed girls had significantly higher (*p* < 0.001) prevalence of ≥90th percentile scores in all performance results in Select, and all except speed in Sport settings compared to Schools testing. No differences were found for either height or body mass across settings (*p* = 0.078 and 0.17, respectively). Boys exhibited smaller differences, with Sport settings yielding significantly higher sprint and agility scores ≥90th percentile (*p* < 0.05), relative to both Schools and Select testing environments. Differences were found for height and body mass across settings (*p* < 0.001 for both analyses, respectively). **Conclusions**: Select environments enhance the identification of physically talented girls, while boys demonstrate broader distribution of performance talent across settings. Findings inform resource allocation for future TID programmes when the primary aim is to maximise the efficiency of finding higher-performance physical talent relative to the number of tests conducted.

## 1. Introduction

Australia, with a population of about 27 million (55th globally) [1], has ranked among the top ten nations for medals in each of the past nine Summer Olympic Games, including three 4th-place and two 6th-place finishes [2]. This success largely stems from significant investment in elite athlete programmes covering scholarships, coaching and training, travel, equipment, and technology innovation [3,4]. To stay competitive internationally, countries like Australia also rely heavily on talent identification (TID) and development programmes [5].

Searching for physically gifted young athletes is laborious, time-consuming, and requires significant financial resources and expertise [3,6,7]. Despite these challenges, it typically involves face-to-face assessment of children and adolescents across physical and anthropometric measures, as well as skill and psychological traits during early talent verification [8]. Nevertheless, this process has become increasingly important, with most of Australia’s Olympians emerging from coordinated TID and development programmes [9,10]. Early talent selection is partly based on the belief in innate ability [11], assuming that early indicators combined with training predict potential for future success [12]. Scientific evidence increasingly supports a genetic basis for physiological talent [13], and elite athletes often report being identified by age 8–11 [14]. However, not all scientists and coaches agree with this approach or the broader TID system [11,15,16].

Several comprehensive reviews on TID in children and adolescents exist [16,17,18,19], generally outlining potential benefits and, importantly, the many pitfalls of current TID methods [20]. Overall, evidence suggests a continuum of innate giftedness in key physical traits (e.g., body size, speed, power, aerobic capacity), making some individuals well-suited to certain sports while most are not suited to high-performance competition [11,21]. Significant but hard-to-quantify interactions among genotypes, environment, and social factors such as nutrition, training, coaching, schooling, location, and parental support further complicate prediction [18]. Non-linear and interindividual differences in development underscore these complexities, making accurate identification of future world-class athletes (placed in the top < 0.001% of the Australian population) at the grassroots level challenging [22,23]. Consequently, many programmes use a relatively low threshold (within the top 2–10%) in initial screening to invite children to the next stage [24]. This is a pragmatic approach endorsed by Australian coaches that balances the need to manage the number of young athletes progressing to the verification stage with the awareness of missing potential talent due to differing rates of development, age effects and maturation stage among other influences at test time. Nevertheless, the success of mass testing is evident in numerous world-class Australian athletes identified through such programmes [9,14,25].

Australia has implemented several major TID programmes over the past four decades [5,25]. A national programme launched in 1994 aimed “to identify and develop potential elite athletes for success at national and international level,” partly targeting the 2000 Sydney Olympics [26]. Using a scientific approach, over 350,000 children aged 12–15 were tested through school visits in the first five years [27], contributing to Australia’s most successful Olympic medal tally [2]. In preparation for the 2032 Brisbane Olympics, Queensland is funding “Youfor32 Talent Search,” described as one of the world’s largest multi-sport TID programmes [28], primarily using a “come-and-try” model. Similarly, South Australia has expanded its “2032 Talent Search” and “Discovering Greatness” programmes, combining school visits with come-and-try testing [29]. These initiatives, supported by substantial national and state funding, aim to identify future Olympians, while also increasing resources for Paralympic talent identification [30].

To date, no empirical studies have determined which testing environment is most effective for identifying physical performance talent. In other words, does screening efficiency vary by setting, where efficiency refers to the number of tests required in order to identify better performers? Three environments were examined: (1) school visits, (2) advertised come-and-try ‘Select’ days, and (3) community sports clubs.

The aim was to calculate the likelihood of participants scoring at or above the 90th percentile in anthropometric or physical performance measures across these settings. We hypothesised that Select settings would capture higher levels of physical talent, as participants with an interest in competitive sport and athletic development would be more likely to self-select into these environments and perform closer to their maximal capacity.

## 2. Materials and Methods

### 2.1. Research Design

The research design was a retrospective, cross-sectional observational study design using physical performance test data collected from children and adolescents across three distinct settings.

### 2.2. Participant Recruitment and Settings

The project analyses physical performance test results from children and adolescents aged 12–17 years, collected between August 2020 and October 2025 across four Australian states. Ages were recorded and rounded down to the last completed year to form 12–17 year bands and analyses were stratified by these bands.

All data were de-identified before analysis; no personally identifiable information was retained, and participants were assigned unique codes for anonymity. Informed consent was obtained from parents and included acceptance of the testing organisation’s terms, which stated that anonymised group results could be used for research. The study was approved by the University of South Australia HREC (ID: 206342) and complied with the Declaration of Helsinki and GDPR data protection requirements.

Physical and performance measures were collected in three settings: (1) school visits (School) during scheduled physical education classes, (2) government-supported “come-and-try” days (Select) promoted to identify potential elite athletes [29], and (3) community sports clubs (Sport) seeking performance data for physical preparation and monitoring. These settings formed the basis for comparing success rates in identifying performance-based talent.

It was not possible to standardise all the testing conditions which occurred across geographic locations, climates, and facilities. For example, the temperature and humidity ranges varied, both indoor and outdoor locations were used, and surfaces used for the aerobic and sprint testing were either indoor timber flooring or outdoor asphalt. Testing was not conducted in the rain or on slippery surfaces. The protocols were consistent for all tests regardless of location. All testers underwent supervised training and test rehearsals to standardise methodologies. They were also provided with a protocol booklet and educational demonstration videos as part of the training process prior to data collection.

School and Sport groups were recruited via email responses to promotions on websites, social media, newsletters for teachers, or word of mouth. Testing occurred in 47 private and 58 public schools, and 11 sports clubs (4 Australian football, 3 hockey, 1 basketball and 3 mixed sports clubs). Participation in individual tests was voluntary, and participants could decline to undertake any component of the testing.

Select sessions were free and advertised on government websites and in regional/state newspapers, while other settings typically involved a service fee for the school or organisation, not participants.

### 2.3. Performance Tests

Physical performance testing followed standardised protocols. The procedure was always conducted in the same order and involved a standardised warm-up of jogging, stretching, and submaximal short-distance movements. Anthropometry measures and performance tests were conducted in a circuit-type fashion to enable the testing team to manage larger groups. Aerobic fitness testing was conducted last. Running shoes were worn for all performance testing.

Anthropometric measures included stature (cm) using a calibrated stadiometer (nearest 1 mm) and body mass (kg) in light clothing using digital scales (nearest 0.1 kg; Seca 813 Electronic, Germany). Measurements were consistently taken without shoes and before other tests and did not involve stretch stature due to staffing limitations. Instructions for stature measurement were to “take a deep breathe in, stand tall with feet together and back of heels touching the wall”.

Handgrip strength (Grip) was measured in a standing position using a hand dynamometer (Takei 5001 Analogue Hand Grip Dynamometer, Takei, Niigata, Japan). An adjustable grip was used, and participants were guided to determine their comfortable grip position. They were instructed to squeeze the dynamometer with maximal force with the right and left hands in turn. Measurements were recorded in 0.5 kg intervals. Duplicate trials were undertaken with at least 2 min recovery time between tests. The final score was the average of the highest values for the left- and right-hand measures [31].

Vertical jump (VJ) is a measure of lower body muscular power. It was measured in 1 cm increments using a countermovement method and a Swift yardstick instrument (https://www.swiftperformance.com/, accessed on 15 April 2026). After determining the stretch height of their outstretched fingers on the lead arm (zero point for jump height determination), participants started in a standing position with both feet on the ground approximately shoulder width apart. They were instructed to take a quick downward movement (countermovement) to a comfortable depth and immediately jump as high as possible in one continuous motion and without a pause at the bottom. Arm swinging was allowed. The best score of 2–3 trials was used in further analysis [32].

A 20 m sprint time (Sprint) is a measure of speed and power. Time from a standing start was measured to the nearest 0.01 s using dual-beam electronic timing gates (https://www.swiftperformance.com/, accessed on 15 April 2026). Runners placed the lead foot immediately behind the start line and were instructed to begin when they felt ready. Sprint time was recorded as the best of 2–3 trials. Recovery time between trials was at least 2 min [33].

A 4 × 10 m agility test (Agility) was undertaken on an “up and back” course between two lines 10 m apart [34]. This consisted of a starting position with the lead foot up to the start line. On the “go” signal, participants accelerated to the 10 m line to pick up a cone placed immediately over the line, followed by a 10 m return to drop the cone over the start line. This was repeated to pick up a second cone and then return to the start as fast as possible. Two trials were conducted and performances were hand-timed by two independent recorders using stop watches to the nearest 0.1 s. The mean value was calculated for each trial and the best performance was used in further analysis. Recovery time between trials was at least 2 min.

Aerobic fitness (Shuttle) was assessed using the maximal multistage 20 m shuttle run test [35]. The test involved running between two lines placed 20 m apart, keeping an incremental pace according to instructions from a pre-recorded audio. The initial speed corresponded to 8.5 km/hr, increasing by 0.5 km/hr each minute [35]. The test stopped when the participants failed to reach the pivot line on two consecutive occasions despite a verbal warning. Each minute equals one stage, so the last completed stage was recorded and converted to a VO_2max_ estimate (mL/kg/min) using the average prediction of two published equations and used in further analysis [35,36].

Anthropometry measures and performance test results were ranked against age- and gender-based population norms that had been assembled from a sample-weighted combination of large representative datasets following a literature search [37,38,39,40,41,42,43]. Any performance test result that was ranked ≥ 90th percentile against the global norms (Table 1) was recognised as a physically talented performance and tallied for further analyses. This threshold was a pragmatic compromise between maintaining a sufficiently broad catchment to minimise the risk of overlooking gifted adolescents and managing the resulting talent pool within the resource constraints of sports institutes. The decision to use global rather than Australian norms was driven by the aim of identifying athletes with the potential for future international high performance, reflecting the level of competition encountered at Olympic and World Championship events.

No maturation-based adjustments were made to the performance data reported in this study. Anthropometry measures were compared to determine if there were any size differences among participants in the three settings. Age-by-setting cell results were only included if the number of participants was >10. Occasionally, not all measures were undertaken by participants due to injury or personal preferences.

### 2.4. Statistics

Distributions of all continuous variables were first examined within each age, gender, and setting using descriptive statistics, including means, variance measures, and skewness values. These descriptive indicators were used to characterise distributional shape and dispersion and to guide subsequent analyses. Pairwise F-tests comparing variance ratios across settings were performed as part of this assessment to examine differences in variability across age groups and settings.

Body mass was the only variable to exhibit non-normal, right-skewed distributions across multiple age groups and settings. Consequently, between-setting differences in the degree of skewness for body mass distributions were examined using a non-parametric bootstrap approach. Bootstrap resampling with replacement was used to generate sampling distributions of skewness estimates for each setting, and differences between settings were evaluated using bias-corrected bootstrap confidence intervals.

Following assessment of distributional characteristics, proportions of participants at or above the 90th percentile were calculated for height, body mass, and individual performance measures by age and gender within each setting, and for combined ages. All analyses were conducted separately for girls and boys. Differences in proportions across settings were assessed using chi-square tests, and statistically significant results were followed by pairwise z-tests for proportions. Bonferroni correction was applied to account for multiple comparisons. Statistical significance was set at *p* < 0.05 unless otherwise stated.

## 3. Results

**Figure 1:**
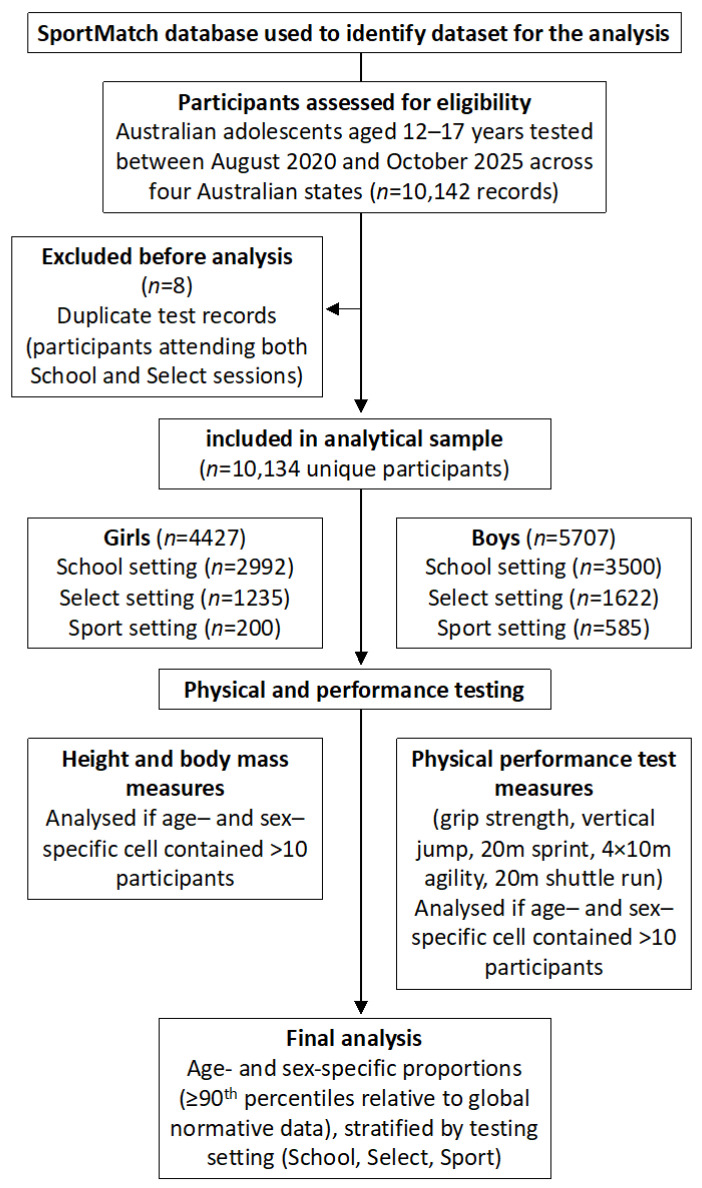
Australian adolescents aged 12–17 years were assessed using standardised anthropometric and physical performance tests across three testing settings: School visits, Select (“come-and-try”) sessions, and community Sport settings, between August 2020 and October 2025. Duplicate test records from participants attending more than one setting were excluded prior to analysis. Participation in individual tests was voluntary. Final analyses involved calculation of age- and sex-specific proportions for each measured variable. Physical talent was defined as performance level at or above the 90th percentile relative to global normative data. Results were stratified by testing setting (School, Select, Sport).

Figure 1 illustrates the breakdown of the 10,134 participants across the three settings. There were no multiple tests or longitudinal analyses included in this study. Table 2 and Table 3 show the number of girl and boy participants who completed each performance test, respectively. Participant ages for both girls and boys were tightly aligned across testing environments within each chronological age band. The between-environment range of mean ages for girls was ≤0.19 years (≤2.2 months) in any band and ≤1.8 months in five of six bands. Within each chronological age band for boys, mean ages were closely aligned across School, Select and Sports environments. The maximum difference between any two environments within a band was ≤0.20 years (≤2.4 months), with five of six bands ≤ 1.9 months. Given the 1-year banding, these differences are trivial for interpretation.

Standard deviations were of similar magnitude across School, Select and Sport settings within each chronological age band for all variables. Pairwise F-tests indicated no evidence of unequal variances (all *p* ≥ 0.053), confirming comparable dispersion across environments in all age bands. The body mass bootstrap analysis confirmed that the observed skewness was stable across resamples, with confidence intervals indicating a consistent and systematic deviation from symmetry across settings. This supported our decision to use the 90th percentile method for body mass, consistent with other variables.

### 3.1. Anthropometry

#### 3.1.1. Girls

The overall mean percentage (95% CI) of height (*n* = 4374) measures that were ≥90th percentile for girls in School, Select and Sport settings were 9.4 (8.4–10.5), 11.1 (9.3–12.9) and 6.7 (3.2–10.2)%, respectively. Chi-square test for girl height showed no difference in proportions across settings (χ^2^ = 5.1; *p* < 0.078). Similarly, the overall mean percentage (95% CI) of body mass (*n* = 4354) measures that were ≥90th percentile for girls in School, Select and Sport settings were 10.3 (9.2–11.4), 8.3 (6.9–10.1) and 8.3 (4.4–12.2)%, respectively. Chi-square test for girl body mass showed no difference in proportions across settings (χ^2^ = 3.5; *p* < 0.17).

#### 3.1.2. Boys

The overall mean percentage (95% CI) of height (*n* = 5620) measures ≥90th percentile for boys in School, Select and Sport settings were 7.0 (6.1–7.8), 7.7 (6.4–9.0) and 14.4 (11.5–17.2)%, respectively. This represents differences of +10.4 and +105.7% for Select and Sport settings relative to the School setting. Similarly, the overall mean percentage (95% CI) of body mass (*n* = 5613) measures ≥90th percentile for boys in School, Select and Sport settings were 11.0 (10.0–12.1), 6.4 (5.2–7.6) and 7.4 (5.2–9.5)%, respectively. This represents differences of −42.1 and −33.2% for Select and Sport settings relative to the School setting.

A chi-square test for boy height showed differences in proportions across settings (χ^2^ = 37.0; *p* < 0.001). Pairwise comparisons showed differences (<0.001) in School versus Sport and Select versus Sport settings (z = −6.0 and −4.7, respectively) but no difference in School versus Select settings (z = −0.9, *p* = 0.38). A chi-square test for boy body mass showed differences in proportions across settings (χ^2^ = 30.7; *p* < 0.001). Pairwise comparisons showed differences (<0.01) in School versus Select and School versus Sport settings (z = 5.2 and 2.7, respectively). There was no difference in participant body mass when Select versus Sport settings were compared (z = −0.82, *p* = 0.42).

### 3.2. Physical Test Performances

The overall mean percentage (95% CI) for the combination of the five performance test results (*n* = 14,944 measures) ≥ 90th percentile for girls in School, Select and Sport settings were 12.6 (12.0–13.2), 21.8 (20.5–23.1) and 17.6 (14.9–20.4)%, respectively. This represents differences of +73.6 and +40.5% for Select and Sport settings, respectively, relative to the School setting. A chi-square test for girls showed differences in proportions across settings (χ^2^ = 192.6; *p* < 0.001). Pairwise comparisons showed differences (<0.001) in School versus both Select and Sport settings (z = −13.8 and −4.0, respectively), and a difference in Select versus Sport groups (z = 2.6, *p* = 0.01).

Chi-square tests conducted on each variable separately showed differences across each of the five test variables: Grip (χ^2^ = 21.8; *p* < 0.001); VJ (χ^2^ = 17.0; *p* < 0.001); Shuttle (χ^2^ = 95.8; *p* < 0.001); Sprint (χ^2^ = 55.2; *p* < 0.001); and Agility (χ^2^ = 12.6; *p* = 0.002). Pairwise comparisons between settings for each test variable are shown in Table 2.

The overall mean percentages (95% CI) for the combination of the five performance test results (*n* = 19,038 measures) ≥ 90th percentile for boys in School, Select and Sport settings were 13.8 (13.2–14.4), 14.6 (13.6–15.9) and 17.3 (15.4–19.2)%, respectively. This represents differences of +5.6 and +25.5% for Select and Sport settings, respectively, relative to the School setting. A chi-square test for boys showed a difference in proportions across settings (χ^2^ = 9.5; *p* = 0.008). Pairwise comparisons showed a difference in a School versus Sport setting (z = −2.75; *p* = 0.006), and no differences in School versus Select (z = −1.92, *p* = 0.054) or Select versus Sport groups (z = −1.4; *p* = 0.16).

**Figure 2:**
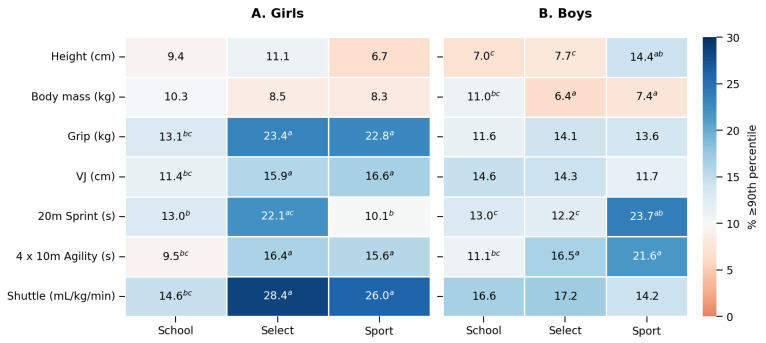
Heat maps showing the proportion (%) of participants achieving performance at or above the 90th percentile relative to global age- and sex-specific normative values across physical fitness tests for girls (**A**) and boys (**B**). Results are shown for three testing settings: School, Select, and Sport. Colour intensity reflects the proportion of participants exceeding the 90th percentile, with darker shading indicating a higher proportion and lighter shading indicating values closer to population expectations. Numeric cell values are displayed as percentages. Superscript letters denote statistically significant differences (*p* < 0.05) relative to specific settings within each test variable and sex: a = different from School, b = different from Select, and c = different from Sport. Cells without superscripts indicate no significant differences between settings.

Chi-square tests conducted on each variable separately showed differences in proportions across Sprint (χ^2^ = 44.7; *p* < 0.001) and Agility (χ^2^ = 25.0; *p* < 0.001) test performances. Pairwise comparisons between settings for these test results are shown in Figure 2.

## 4. Discussion

This study used a large dataset of physical and performance measures from children and adolescents assessed during standardised testing sessions across three settings: (1) Schools during physical education classes, (2) sports institute “Select” come-and-try sessions, and (3) community sports clubs. These are convenience settings in that they are typically used by Australian sports institutes when searching for physical talent. The different settings enabled comparison of physical talent prevalence, or proportion of performance scores above the 90th percentile thresholds, using general population norms for each test. The study aim was achieved and the hypothesis that Select settings would yield the highest level of physically talented participants was supported for the girls but not for the boys.

Results showed some significant gender- and setting-related differences in anthropometric and combined performance characteristics ≥90th percentile of population norms. Among girls, proportions ≥ 90th percentile for height and body mass did not differ by setting. Prior research indicates body size and maturity have limited influence on talent identification in adolescent girls compared to technical and physical performance [44]. It is recognised, however, that this depends on the sport or event—for example, whether it is an open-ended optimisation sport where bigger (e.g., high-jump, basketball) or smaller (e.g., gymnastics, distance running) is advantageous, or a team sport where there might be a mixture of body types, sizes, skills and physiological attributes [45]. Current talent searching priorities in Australia generally involve searching for relatively larger adolescents and/or those with more extreme scores of energy system performances. Specific sport or event choices are determined in subsequent stages of talent verification.

Performance comparisons revealed superior outcomes in Select and Sport settings across all tests versus Schools, except speed in Sport, where sample size was low. These findings highlight the importance of structured talent identification for girls and opportunities to showcase physical ability. Previous studies confirm that systematic programmes, particularly those led by state sporting institutes, effectively identify high-potential athletes and support pathways to elite performance [46]. In this study, talent identification likelihood, based on comparing proportions over the 90th percentile thresholds, was ~70% higher in Select and 47% higher in Sport compared to Schools settings.

Inclusive come-and-try programmes in Select settings provide supportive environments for girls, reducing school-based peer pressures [47,48]. They may also appeal to athletes considering talent transfer between sports based on physiological and skill profiles [46]. However, it is possible that expanding structured training in schools could reduce disparities, improve fitness, and broaden the talent pool [49].

The reasons for performance differences among girls across settings remain unclear but may involve unequal access to training, systematic selection, motivation or a range of sociocultural factors affecting engagement in high-performance environments [47]. While addressing these barriers through inclusive policies, targeted outreach, and equitable resource allocation may equalise opportunities, this was not the focus of the study. The study used the results of a broad base of mass testing adopted by Australian sports institutes when scouting for physically talented boys and girls.

Among boys, extreme value patterns were less pronounced. Taller participants were more prevalent in Sport settings, with a 120% increase over School, suggesting taller boys are more likely to join or be selected for competitive programmes, reflecting the advantage of stature in many boy sports [45]. Conversely, proportions of boys with body mass ≥ 90th percentile were significantly lower in Select and Sport compared to School, indicating heavier mass, shorter stature, and higher BMI may hinder selection or sustained participation. These findings align with evidence that lower relative body mass benefits performance and endurance in youth sport, especially in speed, agility, and aerobic activities [50].

Although Sport showed the highest prevalence of high performers (25.5% above School), overall differences across settings were modest, with only School–Sport comparisons reaching significance, except for agility. This suggests high performance among boys is more evenly distributed and less dependent on selective environments. Physically talented boys are represented across all settings. This pattern may reflect broader opportunities within school programmes, greater habitual engagement in vigorous activity across school and leisure contexts, or a stronger competitive drive to perform well in peer-based environments, rather than talent expressed exclusively within sports settings [51].

The TID approaches and analyses in the current study may not be directly comparable to programmes in other countries. For example, different countries adopt contrasting approaches to TID and athlete development. This reflects differences in sport system organisation, governance, and underlying development philosophies. In Australia, school-based TID initiatives involving anthropometric and physiological screening have been historically implemented and those selected move through to coach-directed, sports institute-based development programmes. Athletes identified through testing in Select or Sport settings may also be redirected into sport-specific pathways through talent transfer initiatives [20,52,53]. In the United Kingdom, TID is more targeted but generally includes school outreach and recruitment through UK sport-funded programmes, including talent transfer initiatives designed to recruit athletes into Olympic sports based on physical characteristics. A system of come-and-try opportunities prioritises measurable physical potential alongside performance history [54]. Germany adopts a club–school hybrid model in which athletes are identified through both club competition and sport-specialised schooling systems. This enables integrated academic and sporting development and ongoing monitoring of talent progression and generally reflects broader European models where early detection and career development are prioritised while maintaining participation breadth [20,55]. In contrast, China and Russia use more centralised systems characterised by early identification through early school-based testing and selected talent moving into specialised sports schools for intensive training [56]. The United States relies on a decentralised model in which talent emerges through school sport and collegiate competition rather than formalised national screening systems [57]. In other models, particularly in sports such as football in European and South American nations, academy-based systems are embedded within professional clubs. These play a central role in early identification and long-term, sport-specific athlete development.

Collectively, these systems reflect fundamentally different philosophies regarding the timing, mechanisms, and structure of talent identification in elite sport development [58].

### Limitations

This study has several limitations: (1) School and Sport group selection was non-random, potentially biasing results toward certain sports or schools. For example, height benefits sports like Australian football, netball, and basketball but less so for hockey. (2) School testing included urban and rural locations, but Sport testing was urban only; Select sessions were metropolitan and often required parental transport, all potentially impacting the cohort tested. (3) Sport participants were relatively few, so small changes in 90th percentile counts disproportionately affected percentages. Results were combined to reduce this effect, but limitations remain: (4) The 90th percentile thresholds were based on published datasets, which may not reflect global norms. However, given the large original sample, they likely approximate developed-world norms and our comparisons used consistent reference values. (5) This was not a cost–benefit analysis, but school visits are clearly more expensive than centralised testing, especially for rural schools. (6) It is unknown how many School or Select participants also played community sport, though likely substantial in the Australian context. (7) Performance measures were not adjusted for maturation or relative age, as raw scores were the focus in the mass screening phase of physical talent detection. (8) Finally, differences in environmental conditions during the testing were not used to adjust scores, although it is recognised that temperature, air-density, floor surface type, and other factors may impact performances.

## 5. Conclusions

Finding physical talent among children and adolescents is influenced by setting and gender, with structured Select and Sport programmes enhancing talent identification in girls, while boys demonstrate more homogeneous performance across settings. These results are contextual in that they reflect the current broad-based screening processes adopted across much of the Australian TID landscape. Within this general approach for identifying physically talented boys and girls, there is a greater probability of finding talent among girls in state-based sports institute come-and-try test sessions. That is, it appears the efficiency of finding physically talented girls is greater in Select settings compared to the other settings. For boys, it appears physical talent emerges across all settings in a more uniform way. Practically, the findings indicate that physically talented adolescents are present across all testing environments. However, the higher concentration of talented girls in Select settings suggests that, when talent identification is the primary objective, Select environments offer greater efficiency in terms of resource allocation and maximise coach attendance and their added experience in talent selection and development. Similarly, community Sport settings may be the most effective context for identifying physically talented boys. In contrast, when the dual aims are talent identification and the promotion of physical literacy and educational engagement, School settings may be the preferred option. Future research may explore the psychological characteristics of athletes who attend Select sessions, compare attrition rates across pathways, and use historical data from elite athletes to retrospectively examine their early-life test performance percentile rankings.

## Figures and Tables

**Table 1:** The 90th percentile thresholds for anthropometric and performance tests (12–17 years) for girls (top) and boys (bottom).

**Girls 90th Percentile Thresholds**						
Age (yr)	12	13	14	15	16	17
Height (cm)	169.5	172.0	173.8	174.0	174.4	174.6
Body mass (kg)	63.6	67.1	69.8	71.9	73.4	76.3
Grip (kg)	29.7	32.7	34.9	36.2	37.3	38.2
VJ (cm)	43.6	44.9	46.5	47.5	48.0	48.6
20 m Sprint (s)	3.57	3.48	3.43	3.34	3.30	3.28
4 × 10 m Agility (s)	10.46	10.28	10.23	10.16	10.12	10.03
Shuttle (mL/kg/min)	48.2	48.3	48.5	48.8	49.7	50.6
**Boys 90th Percentile Thresholds**						
Age (yr)	12	13	14	15	16	17
Height (cm)	171.2	179.1	184.4	186.6	188.2	189.4
Body mass (kg)	62.9	70.3	75.1	80.6	85.9	87.5
Grip (kg)	34.1	41.2	46.9	50.0	53.6	56.0
VJ (cm)	48.0	51.9	57.1	60.7	63.5	63.6
20 m Sprint (s)	3.34	3.22	3.10	3.07	3.04	3.03
4 × 10 m Agility (s)	10.13	9.71	9.45	9.39	9.29	9.20
Shuttle (mL/kg/min)	54.7	56.8	57.5	59.6	60.0	61.2

**Table 2:** Proportion of girl participants ≥ 90th percentile for individual performance tests is shown by age subgroup and setting. Participant numbers for each setting and age group appear along the bottom. Cells with fewer than 10 participants were excluded from analysis and left blank.

Girls % ≥ 90th %’ile	12	13	14	15	16	17	Total *n* ≥ 90th %’ile	Total Participants
Height								
School	6.4	9.4	8.4	12.1	11	6.9	279	2962
Select	22.8	7.3	8.5	16	13.2	11.3	135	1212
Sport	0	6.1	12	2.2	6.7		13	193
Body mass								
School	13.7	9.6	10.9	8.1	12.3	13.8	302	2942
Select	24.6	6.1	7.3	9.6	6.6	12.7	103	1212
Sport	0	9.1	10.7	8.9	6.7		16	193
Grip								
School	14.6	13.7	14.3	11.3	4.2		178	1358
Select	33.3	14.3	36.7	14.3	21.4		47	201
Sport	36.4	27.8	19.4	23.7	20		34	149
VJ								
School	12.6	10.8	10.1	11.6	20.6	18.5	322	2815
Select	24.6	19	13	11.1	16.7	22.5	191	1203
Sport	8	15.6	16.7	20.5	21.4		31	187
Shuttle								
School	14.4	15.9	13.2	15.7	13.4	11.1	334	2282
Select	29.2	32.5	34.9	19.2	22.5	22.4	329	1158
Sport	28	46.7	22.7	16	23.1		26	100
20 m Sprint								
School	11.9	12.7	16.2	10.2	6.9	12	341	2627
Select	28.1	22.3	29	18.3	17.6	5.6	265	1199
Sport	10	10.3	11.5	7.1			14	138
4 × 10 m Agility								
School	8.7	9.8	7.3	11.9	10.5		107	1127
Select	5.4	12.8	28.8	22.6	7.1		36	220
Sport	36.4	16.7	12.5	9.3	15.4		28	180
Participant numbers								
School	256	934	1003	623	146	30		2992
Select	60	349	345	256	151	74		1235
Sport	25	33	75	45	15	7		200

**Table 3:** Proportion of boy participants ≥ 90th percentile for individual performance tests is shown by age subgroup and setting. Participant numbers for each setting and age group appear along the bottom. Cells with fewer than 10 participants were excluded from analysis and left blank.

Boys % ≥ 90th %’ile	12	13	14	15	16	17	Total *n* ≥ 90th %’ile	Total Participants
Height								
School	12.3	5.4	4.6	8.5	9.3	5	241	3452
Select	14.3	7.5	6.2	6.7	9.9	8.7	122	1583
Sport	6.3	6.9	5.4	15.5	22.8	14	84	585
Body mass								
School	13.9	9.7	12.1	11.7	5.4	13.3	380	3446
Select	11.1	4	6.6	7	6.3	8.7	101	1583
Sport	6.3	13.8	2.2	10.3	3.7	9.8	43	584
Grip								
School	16.2	8.9	14.3	11.1	8	10.7	200	1724
Select	2.6	19.4	11.3	6.7	23.3	23.5	46	327
Sport		0	20	3.6	26.3		24	177
VJ								
School	11.2	15.1	14.9	16.4	12.8	10.3	483	3300
Select	18	13.9	9.7	13.8	16.4	24.1	224	1570
Sport	6.3	11.1	17.4	16.1	8.3	10	66	562
Shuttle								
School	21.7	16.4	16.5	17.3	10.4	7.7	446	2683
Select	26.4	19.6	17.7	16.3	10.5	17.5	246	1432
Sport	6.3	24	8.7	13.3	7.4	28.6	17	120
20 m Sprint								
School	9.6	11.3	13	12.3	23.3	20.4	396	3050
Select	6.3	8.2	11.2	6.6	20.3	28.6	190	1558
Sport		7.7	38	19	15.3	26.7	113	476
4 × 10 m Agility								
School	10.5	8.8	11.2	14.2	11	11.1	157	1410
Select	13.7	16.3	16.3	10.7	21.3	38.9	63	381
Sport	0	3.8	22.2	17.7	32.6	37.9	58	268
Participant numbers								
School	388	1052	982	761	257	60		3500
Select	64	429	405	340	227	157		1622
Sport	16	29	92	97	136	215		585

## Data Availability

The original contributions presented in this study are included in the article. Further inquiries can be directed to the corresponding author.

## References

[1] Worldometer Population by Country. https://www.worldometers.info/world-population/population-by-country/.

[2] International Olympic Committee Olympic Medal Tables. https://www.olympics.com/en/olympic-games/olympic-results.

[3] Hogan K., Norton K. (2000). The ‘price’ of Olympic gold. J. Sci. Med. Sport.

[4] Commonwealth Department of Health and Aged Care Budget 2024–2025 Overview 2024. https://www.health.gov.au/sites/default/files/2024-05/budget-2024-25-budget-overview.pdf.

[5] Weissensteiner J.R. (2023). The global evolution of talent promotion within Olympic sports: A focus on the national systems and contribution of the former German Democratic Republic, Australia, and the United Kingdom. Front. Sports Act. Living.

[6] Larkin P., Reeves M.J. (2018). Junior-elite football: Time to re-position talent identification?. Soccer Soc..

[7] UK Sport (2015). Annual Report and Accounts 2014–2015.

[8] Australian Institute of Sport FTEM framework. Canberra (ACT): Australian Institute of Sport. https://www.ausport.gov.au/ais/ftem.

[9] News Limited (2000). 2000 Australian Olympic Team Handbook and Media Guide.

[10] Hoare D. (1998). Talent Search: A Review and Update. Sports Coach.

[11] Baker J., Wattie N. (2018). Innate talent in sport: Separating myth from reality. Curr. Issues Sport Sci..

[12] Howe M.J.A., Davidson J.W., Sloboda J.A. (1998). Innate talents: Reality or myth?. Behav. Brain Sci..

[13] Antero J., Saulière G., Marck A., Toussaint J.-F. (2018). A medal in the Olympics runs in the family: A cohort study of performance heritability in the games history. Front. Physiol..

[14] Gulbin J., Gagne F., Weissensteiner J.R. (2010). A look through the rear view mirror: Developmental experiences and insights of high performance athletes. Talent. Dev. Excell..

[15] Sedeaud A., Difernand A., De Larochelambert Q., Irid Y., Fouillot C., du Sel N.P., Toussaint J.-F. (2025). Talent identification: Time to move forward on estimation of potentials? Proposed explanations and promising methods. Sports Med..

[16] Till K., Baker J. (2020). Challenges and possible solutions to optimizing talent identification and development in sport. Front. Psychol..

[17] Johnston K., Wattie N., Schorer J., Baker J. (2018). Talent Identification in Sport: A Systematic Review. Sports Med..

[18] Leite N., Calvo A.L., Calleja-Gonzalez J., Gonçalves B., Cumming S. (2022). Talent Identification and Development in Sports Performance.

[19] Rongen F., McKenna J., Cobley S., Till K. (2018). Are youth sport talent identification and development systems necessary and healthy?. Sports Med. Open.

[20] Vaeyens R., Lenoir M., Williams A.M., Philippaerts R.M. (2008). Talent identification and development programmes in sport: Current models and future directions. Sports Med..

[21] Baker J., Wattie N., Schorer J. (2019). A proposed conceptualization of talent in sport: The first step in a long and winding road. Psychol. Sport. Exerc..

[22] Gulbin J., Weissensteiner J., Oldenziel K., Gagné F. (2013). Patterns of performance development in elite athletes. Eur. J. Sport. Sci..

[23] Leite N., Calvo A.L., Cumming S., Gonçalves B., Calleja-Gonzalez J. (2021). Editorial: Talent Identification and Development in Sports Performance. Front. Sports Act. Living.

[24] Ziemainz H., Gulbin J. (2002). Talent selection, -identification and -development exemplified in the Australian Talent Search Programme. New Stud. Athl..

[25] Hoare D., Warr C. (2000). Talent identification and women’s Soccer: An Australian experience. J. Sports Sci..

[26] Australian Sports Commission (1994). Sport Search, the Search is Over: Norms for Sport Related Fitness Tests in Australian Students Aged 12–17 Years.

[27] Tomkinson G.R., Olds T.S., Gulbin J. (2003). Secular trends in physical performance of Australian children: Evidence from the Talent Search program. J. Sports Med. Phys Fit..

[28] Queensland Government Youfor32 Sports Initiative. https://www.qasport.qld.gov.au/youfor2032.

[29] South Australian Government Talent Search Initiative. https://www.sasi.sa.gov.au/about/talent-search.

[30] Australian Government (2024). Historic Investment in High Performance Sport.

[31] Laurson K.R., Saint-Maurice P.F., Welk G.J., Eisenmann J.C. (2017). Reference curves for field tests of musculoskeletal fitness in U.S. Children and Adolescents: The 2012 NHANES National Youth Fitness Survey. J. Strength Cond. Res..

[32] Warr D.M., Pablos C., Sánchez-Alarcos J.V., Torres V., Izquierdo J.M., Carlos Redondo J. (2020). Reliability of measurements during countermovement jump assessments: Analysis of performance across subphases. Cogent Soc. Sci..

[33] Haugen T., Buchheit M. (2016). Sprint running performance monitoring: Methodological and practical considerations. Sports Med..

[34] Ortega F.B., Artero E.G., Ruiz J.R., Vicente-Rodriguez G., Bergman P., Hagströmer M., Ottevaere C., Nagy E., Konsta O., Rey-López J.P. (2008). Reliability of health-related physical fitness tests in European adolescents. The HELENA Study. Int. J. Obes..

[35] Léger L.A., Mercier D., Gadoury C., Lambert J. (1988). The multistage 20 metre shuttle run test for aerobic fitness. J. Sports Sci..

[36] Barnett A., Chan L.Y., Bruce L.C. (1993). A preliminary study of the 20-m multistage shuttle run as a predictor of peak VO_2_ in Hong Kong Chinese students. Pediatr. Exerc. Sci..

[37] Fryar C., Gu Q., Ogden C. (2012). Anthropometric Reference Data for Children and Adults: United States, 2007–2010.

[38] Iglesias-Soler E., Rúa-Alonso M., Rial-Vázquez J., Lete-Lasa J.R., Clavel I., Giráldez-García M.A., Rico-Díaz J., Corral M.R.-D., Carballeira-Fernández E., Dopico-Calvo X. (2021). Percentiles and Principal Component Analysis of Physical Fitness From a Big Sample of Children and Adolescents Aged 6–18 Years: The DAFIS Project. Front. Psychol..

[39] Silverman I. (2015). Age as a moderator of the secular trend for grip strength in Canada and the United States. Ann. Hum. Biol..

[40] Tomkinson G.R., Carver K.D., Atkinson F., Daniell N.D., Lewis L.K., Fitzgerald J.S., Lang J.J., Ortega F.B. (2018). European normative values for physical fitness in children and adolescents aged 9–17 years: Results from 2,779,165 Eurofit performances representing 30 countries. Br. J. Sports Med..

[41] Tomkinson G.R., Lang J.J., Tremblay M.S., Dale M., LeBlanc A.G., Belanger K., Ortega F.B., Léger L. (2017). International normative 20 m shuttle run values from 1,142,026 children and youth representing 50 countries. Br. J. Sports Med..

[42] Zhang F., Yin X., Bi C., Li Y., Sun Y., Zhang T., Yang X., Li M., Liu Y., Cao J. (2020). Normative Reference Values and International Comparisons for the 20-Metre Shuttle Run Test: Analysis of 69,960 Test Results among Chinese Children and Youth. J. Sports Sci. Med..

[43] Ruiz J.R., Castro-Pinero J., Espana-Romero V., Artero E.G., Ortega F.B., Cuenca M.M., Jimenez-Pavon D., Chillon P., Girela-Rejon M.J., Mora J. (2010). Field-based fitness assessment in young people: The ALPHA health-related fitness test battery for children and adolescents. Br. J. Sports Med..

[44] Malina R.M., Cumming S.P., Rogol A.D., Coelho-E-Silva M.J., Figueiredo A.J., Konarski J.M., Kozieł S.M. (2015). Bio-banding in youth sports: Background, concept, and application. Sports Med..

[45] Norton K., Olds T. (2001). Morphological evolution of athletes over the 20th century: Causes and consequences. Sports Med..

[46] Cobley S., Schorer J., Baker J. (2012). Talent Identification and Development in Sport: International Perspective.

[47] Slater A., Tiggemann M. (2011). Gender differences in adolescent sport participation, teasing, self-objectification and body image concerns. J. Adolesc..

[48] Cockburn C., Clarke G. (2002). Everybody’s looking at you!: Girls negotiating the ‘femininity deficit’ they incur in physical education. Womens Stud. Int. Forum.

[49] Dobbins M., Husson H., DeCorby K., LaRocca R.L. (2013). School-based physical activity programs for promoting physical activity and fitness in children and adolescents aged 6 to 18. Cochrane Database Syst. Rev..

[50] Artero E.G., España-Romero V., Ortega F.B., Jimenez-Pavon D., Ruiz J.R., Vicente-Rodriguez G., Bueno M., Marcos A., Gómez-Martinez S., Urzanqui A. (2010). Health-related fitness in adolescents: Underweight, and not only overweight, as an influencing factor. The AVENA study. Scand. J. Med. Sci. Sports.

[51] Kretschmer L., Salali G.D., Andersen L.B., Hallal P.C., Northstone K., Sardinha L.B., Dyble M., Bann D., International Children’s Accelerometry Database (ICAD) Collaborators (2023). Gender differences in the distribution of children’s physical activity: Evidence from nine countries. Int. J. Behav. Nutr. Phys. Act..

[52] Abbott A., Collins D. (2004). Eliminating the dichotomy between theory and practice in talent identification and development: Considering the role of psychology. J. Sports Sci..

[53] Vaeyens R., Güllich A., Warr C.R., Philippaerts R. (2009). Talent identification and promotion programmes of Olympic athletes. J. Sports Sci..

[54] Collins D., MacNamara Á (2012). The rocky road to the top: Why talent needs trauma. Sports Med..

[55] Güllich A. (2014). Many roads lead to Rome—Developmental paths to Olympic gold in men’s field hockey. Eur. J. Sport Sci..

[56] Green M., Houlihan B. (2005). Elite Sport Development: Policy Learning and Political Priorities.

[57] Baker J., Schorer J., Wattie N. (2018). Compromising Talent: Issues in Identifying and Selecting Talent in Sport. Quest.

[58] Platvoet S.W.-J., van Heuveln G., van Dijk J., Stevens T., de Niet M. (2023). An early start at a professional soccer academy is no prerequisite for World Cup soccer participation. Front. Sports Act. Living.

